# Biomechanics of flail chest injuries: tidal volume and respiratory work changes in multiple segmental rib fractures

**DOI:** 10.1007/s00068-024-02754-x

**Published:** 2025-01-17

**Authors:** Julian N. Zierke, Georg N. Duda, Karl F. Braun, Vera Jaecker, Ulrich Stöckle, Philipp Damm, Mark Heyland, Marcel Niemann

**Affiliations:** 1 Julius Wolff Institute, Berlin Institute of Health at Charité—Universitätsmedizin Berlin, Augustenburger Platz 1, 13353 Berlin, Germany; 2 Department of Trauma Surgery, University Hospital Rechts Der Isar, Technical University of Munich, Ismaninger Str. 22, 81675 Munich, Germany; 3 Center for Musculoskeletal Surgery, Charité—Universitätsmedizin Berlin, corporate member of Freie Universität Berlin and Humboldt-Universität zu Berlin, Augustenburger Platz 1, 13353 Berlin, Germany

**Keywords:** Rib fractures, Finite element model, Paradoxical chest wall movement, Surgical stabilization of rib fractures, Pulmonary function, Multiple trauma, Respiratory insufficiency

## Abstract

**Background:**

Flail chest (FC) injuries are segmental osseous injuries of the thorax that typically result from high-energy blunt trauma and regularly occur in multiple trauma (MT) patients. FC injuries are associated with paradoxical chest wall movements and, thus, have a high risk of respiratory insufficiency or even death. An increasing number of studies recommend an early surgical stabilization of FC injuries, but a definite trigger that would indicate surgery has, thus far, not been identified.

**Methods:**

Based on real-world injury computed tomography (CT) data, this study aimed to establish a finite elements (FE) model of a thorax simulating spontaneous breathing. The model is based on a 0.625 mm slice thickness CT data set. In this FE model, various FC injury patterns were implemented to examine the impact of an increasingly large flail segment on tidal volume and respiratory work. The impact of the segmental defect sizes on the outcome measures mentioned above was examined using correlation analyses.

**Results:**

The FE model in this study reliably simulated the spontaneous breathing patterns of an actively breathing patient in an uninjured setting as a reference and showed clinically realistic movements of the flail segments for various injury settings. Correlation analysis showed a significant negative correlation between the FC size and tidal volume (R^2^ = 0.852, *p* = 0.003), while absolute (R^2^ = 0.845, *p* = 0.0096) and relative loss (R^2^ = 0.844, *p* = 0.0096) of tidal volume concerning the intact model and the compensatory respiratory work required (R^2^ = 0.816, *p* = 0.0136) were positively correlated with FC size.

**Conclusion:**

This study presents an FE model of the thorax of a patient who presented to our clinic as an MT patient with an FC injury. The FE model fulfills physiologic active breathing patterns and simulates an FC injury’s paradoxical movement, realistically depicting clinical observations. The FE model showed that the number of consecutive ribs involved in the flail segment and the length of the flail segment significantly impacted active breathing concerning tidal volumes and respiratory work. With this, we have made the first step to define a trigger for surgery.

**Supplementary Information:**

The online version contains supplementary material available at 10.1007/s00068-024-02754-x.

## Introduction

Flail chest (FC) refers to a severe injury of the osseous thorax. This injury typically results from a high-energy trauma [1–3] and leads to an unstable segment of the bony thorax. In uninjured thoraces, thoracic excursion for spontaneous breathing mainly occurs through the diaphragm’s and intercostal muscles’ contraction [4, 5]. During inspiration, an increase in intrathoracic volume creates a negative intrapleural pressure relative to the surrounding atmospheric pressure, causing air to flow into the alveoli along the pressure gradient [4]. In FC injuries, part of the chest wall is mechanically detached. As a result, the fractured segment will collapse inward against the rest of the thorax during inspiration due to negative intrathoracic pressure, and it will be pushed outward during expiration due to increased intrathoracic pressure. This has been described as paradoxical chest wall movements [6, 7], usually associated with severe pain [8, 9].

Thus far, there is a consensus about the radiographic morphology of a flail segment (FS). An FS is commonly defined as segmental fractures of at least three contiguous ribs [6]. However, this solely addresses the morphology of the underlying injury pattern and does not address the possibly resulting paradoxical chest wall movements. Accordingly, there is yet no consensus about the required number of contiguously fractured ribs necessarily resulting in the clinical manifestation of a FC [6].

Affected patients have a high risk of pulmonary infections, with over 80% requiring intensive medical care and nearly 60% needing invasive ventilation [2]. Almost a quarter of the affected patients die as a result of a FC injury [1, 8, 9].

Aiming to reduce mortality and avoid secondary complications related to prolonged length of stay (LOS), various authors examined surgical stabilization of FC injuries [10–12]. A recent meta-analysis concluded that early FC stabilization can lead to a lower incidence of pneumonia, shorter LOS, and shorter invasive ventilation [13]. On the other hand, conservative treatment can also deliver satisfactory results in some patients. With this, complications associated with surgery can be avoided [14, 15].

MT patients are highly vulnerable in the acute phase after trauma, and concomitant injuries commonly need to be addressed. Accordingly, identifying patients at risk for respiratory complications due to thoracic injury is paramount in deciding on the most promising treatment concept. Thus far, the indication for surgery is most often based on the radiographic proof of a FC injury. However, FC injuries were defined without evaluating the biomechanics implications.

To overcome this pitfall, we have included an evaluation of mechanical tissue strains and paradoxical breathing in patients, depending on the biomechanical injury profile. Specifically, we aimed to establish a finite element (FE) analysis of real-world injury data to infer the effect of the size of different FC patterns on respiration and evaluate their consequences on tissue straining and biomechanical levels. With this, we aimed to gain a further understanding of the impact of the flail pattern on breathing physiology and help guide clinical decision-making in such injuries on a validated biomechanical basis that complements the current clinical staging.

## Methods

Approval from the institutional review board was obtained before data acquisition (Ethikkommission der Charité—Universitätsmedizin Berlin, application number EA4/119/20). Our clinic’s electronic patient chart system (SAP ERP 6.0 EHP4, SAP AG, Walldorf, Germany) was used to select the patient cohort. We solely focused on multiple trauma (MT) patients with a concomitant FC injury. The definite selection protocol of eligible patients was previously reported [16]. Digital Imaging and Communications in Medicine (DICOM) data sets of postoperatively assessed computed tomography (CT) images were exported from Visage^®^ (Visage^®^ for Microsoft Windows, Version 7.1, Visage Imaging, Inc., Pro Medicus Limited, Richmond, Australia).

We chose one representative patient with an estimated body weight of around 100 kg from a pool of 11 MT patients with FC injuries. We modeled chest expansion as a reversible pressure change in a homogenized surface shell lung model, similar to a rubber balloon. Lung expansion can be caused by pressure increase (e.g., air inflow, external pressure ventilation) or thorax expansion. First, we modeled the expansion of the lung by applying an overpressure (e.g., passive ventilation) into an intact thorax model. We used the resulting displacement of the thorax as a boundary condition to model the active ventilation. Modeling was performed with the following steps:

### Simulation part 1: passive ventilation

1. A consistent unit system with the following units was set (Table 1). Modeling was performed using Abaqus/CAE 2019 (Dassault Systèmes Simulia Corp. 2018, Johnston, RI, USA).
2. Physical constants: the universal gas constant was set at 8.31434 m^3^⋅Pa⋅K^−1^⋅mol^−1^, and the absolute temperature was set at 0 K.
3. The geometry of the lung was defined as a shell in 3D from a representative patient CT. The shell was meshed with linear triangular S3R elements (#nodes = 26,483, #elements = 52,962). Since the lung will be the contact slave surface, it is finer meshed.
4. Hyperelastic material properties were assigned to the lung shell with a Mooney–Rivlin material model with coefficients of C10 = 580 kPa, C01 = 580 kPa, D1 = 0, and a shell thickness of 3 mm.
5. The FLUID CAVITY interaction model was set with a reference point inside the lung to simulate a fluid cavity within the lung’s inner surface. The fluid within the lung was air (pneumatic definition) with an ideal gas molecular weight of 0.0289647 kg/mol and a temperature of 309.15 K (36 °C). Ambient pressure was set at 101,325 Pa.
6. The first calculation step (lung shell expansion) was created with non-linear geometry and a starting increment length of 0.001 [0.1%], a maximum increment length of 1 [100%], and a minimum increment length of 1E-07 [0.00001%].
7. Boundary conditions were defined at the trachea (i.e., ENCASTRE boundary condition: U1 = U2 = U3 = UR1 = UR2 = UR3 = 0; fully fixed in space) and for a pressure increase within the lung (15 mmHg ≈ 2 kPa overpressure) [17].
8. The lung shell was expanding homogeneously. History output at the reference point was requested with hydrostatic fluid pressure within the lung shell (PCAV: Pressure in the CAVity) and hydrostatic fluid cavity volume (CVOL: Cavity VOLume).
9. A bony thorax geometry was created with solid quadratic tetrahedral C3D10 elements (#nodes = 397,918, #elements = 246,840).
10. Bone material properties were defined by Young’s modulus of 0.5 GPa and Poisson’s ratio of 0.3.
11. The bony thorax was fixed via a boundary condition at the spine (both encastred, i.e., fully fixed in space).
12. A surface-to-surface contact interaction was defined between the thorax (master) and the expanding lung shell elements (slave), representing the pleura interaction. The contact properties had a tangential behavior with a friction coefficient of 0.3 (penalty formulation) and normal behavior of exponential pressure-overclosure of 0.035 kPa at 0 mm clearance and 0 kPa at 0.5 mm clearance.
13. Displacement of the bony thorax and lung was measured at all nodes during the first step through expansion of the lung shell pushing onto the ribs.
14. Spirometry data suggest regular tidal volumes to be around 6–8 ml/kg for ideal body weight, the generally accepted recommendation for lung-protective ventilation [18, 19]. Based on the estimated body weight of the patient this model is based on, we verified the tidal volume to be around 700 ml and the cranial and ventral movement at the sternum (fifth costal cartilage) to be in a physiological range of 3–5 mm [20].

**Table 1:** Consistent unit system chosen for modeling

Mass unit	Kilogram	[kg]
Length unit	Meter	[m]
Time unit	Second	[s]
Force unit	Newton	[N]
Pressure unit	Pascal	[Pa]
Density unit	Mass / Length^3^	[kg/m^3^]
Temperature unit	Kelvin	[K]
Element numbers (amount of substance)	Mole (Avogadro number)	[mol]

### Simulation part 2: active inspiration in an intact thorax

**Fig. 1:**
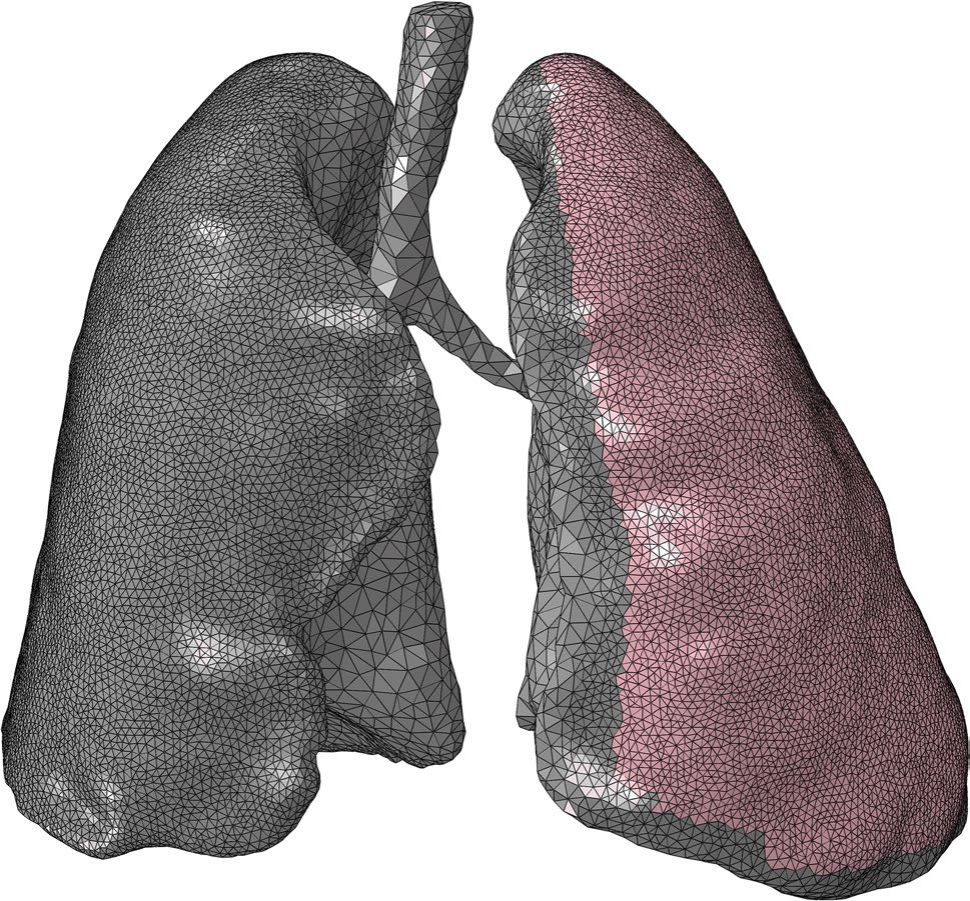
Material assignments of the lung tissue. Stiffer material is indicated in grey, and softer material is indicated in pink

1. The second calculation step was created with non-linear geometry and starting increment length of 0.01, maximum increment length of 0.1, minimum increment length of 1E-10, and activated automatic adaptive stabilization with a damping factor of 2E-07 and maximal stabilization ratio to strain energy of 0.05.
2. The parts from the simulation of passive ventilation were used.
3. The bone material was unchanged, but the hyperelastic material parameters of the lung were adjusted. There was one stiff variant with C10 = 500 kPa, C01 = 500 kPa, and D1 = 0, and a softer variant with C10 = 1 kPa, C01 = 0.17 kPa, and D1 = 0. The stiffer variant was assigned to all lung elements outside the potential fracture side of the left ribs. To prevent numerical instabilities, the soft variant was assigned to the elements at the fracture side beneath the potentially fractured ribs (Fig. 1). The Mooney–Rivlin material parameters were approximated from linear elastic properties as follows [21, 22]:
  $$c_{10} + c_{01} = \frac{E}{{4\left( {1 + v} \right)}}$$
  $$with\,c_{10} = c_{01}$$
  $$c_{10/01} = \frac{E}{{8\left( {1 + v} \right)}} = \frac{5\,kPa}{{8\left( {1 + 0.45} \right)}} = 0.43\,kPa$$
  ${c}_{10}$: Mooney-Rivlin 2-parameter model, material constant ${c}_{10}$
  ${c}_{01}$: Mooney-Rivlin 2-parameter model, material constant ${c}_{01}$
  E: Young’s modulus
  ν: Poisson’s ratio

As the material parameters for lung tissue vary in the literature [22], we have iteratively adjusted the calculated values for c10 and c01 to obtain a numerically stable simulation.

**Fig. 2:**
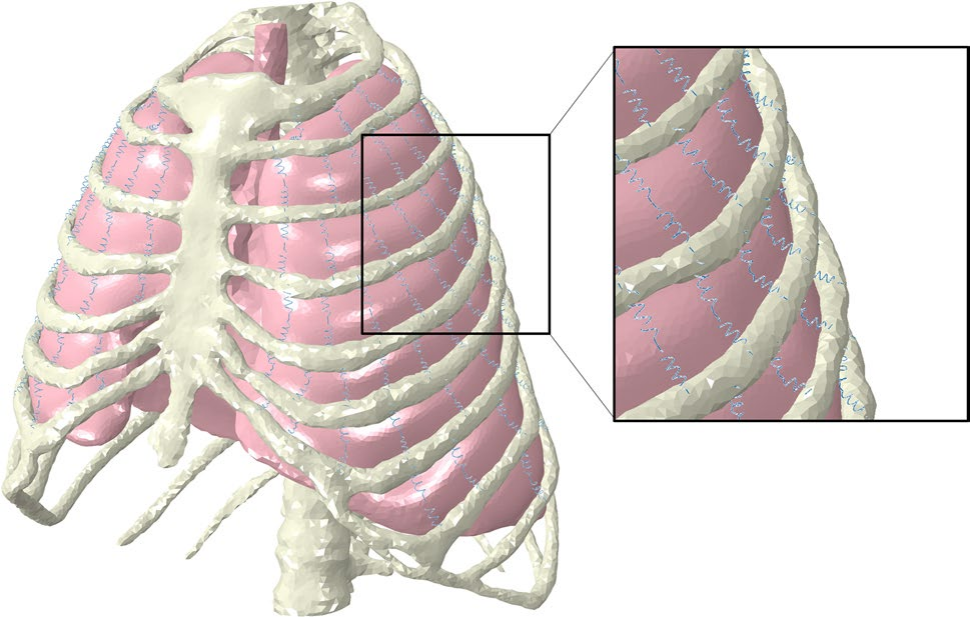
Assembly consisting of the bony thorax, lung, and intercostal springs

4. Linear elastic springs with a stiffness of 10,000 N/m were inserted into the intercostal area at regular intervals, connecting the thorax with lung nodes to simulate the lung’s attachment to the intercostal muscles (Fig. 2).
5. The lung surface near the ribs was tied to the bony thorax. This allowed the lung surface to follow the movement of the thorax.
6. The measured 3D thoracic displacement from previous passive ventilation was applied to all thoracic nodes. The measured lung displacement was applied to a selected lung region only in the mediolateral direction and to the diaphragm in the caudocranial direction. This results in a more stable simulation while reducing the resulting tidal volume change within a physiological range of approximately 400 ml. The fluid cavity pressure was changed to − 0.3 kPa (− 2.2 mmHg), simulating active inspiration [23].
7. Changes in volume and pressure were measured during the entire simulation.

### Simulation part 3: active inspiration fractured thorax

**Fig. 3:**
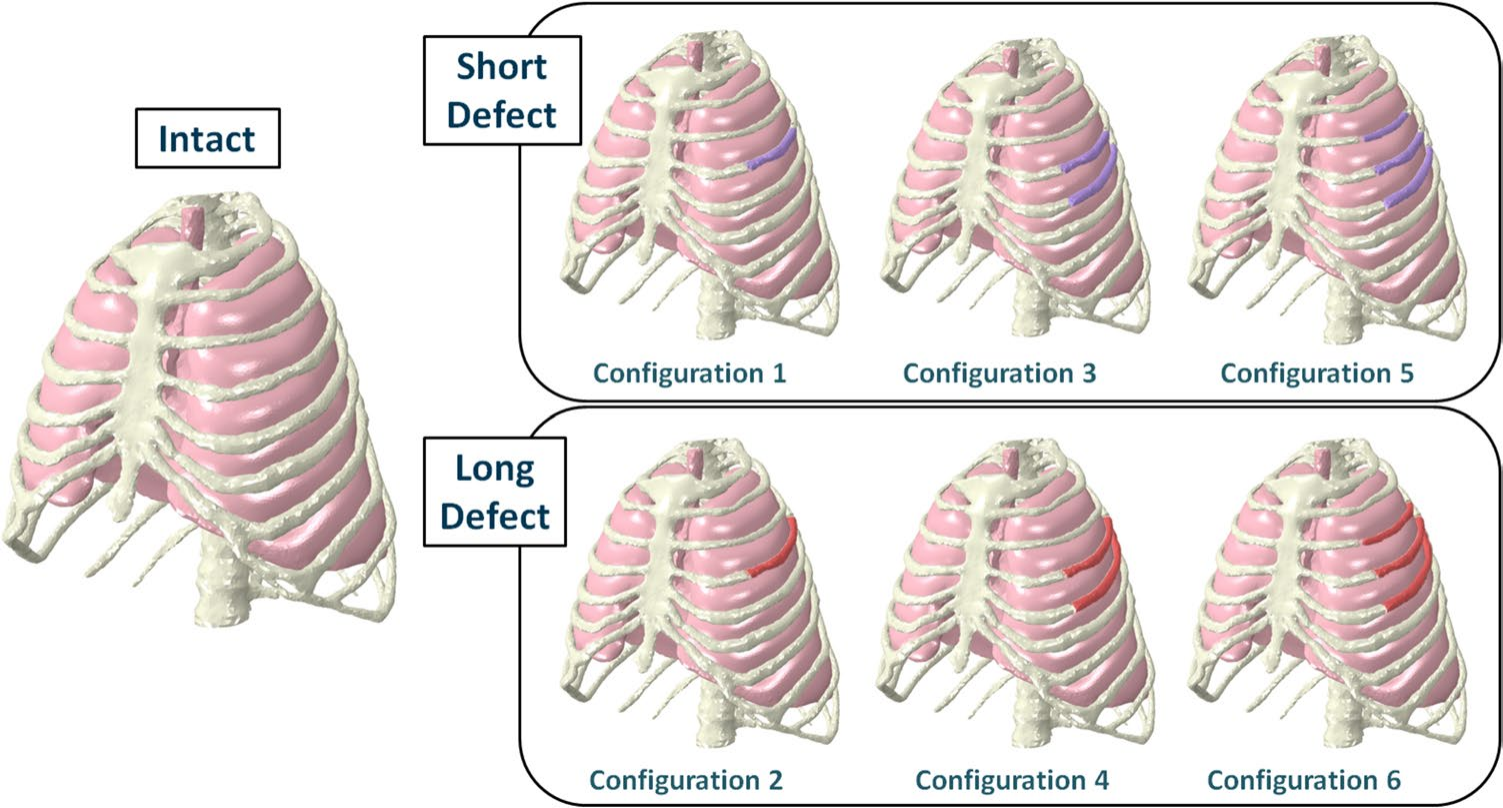
Thorax models of segmental injury for the different configurations of intact (left), one, two, or three consecutively fractured ribs (right), with a short (top in purple) or a long defect length (bottom in red). Configuration 1: Segmental fracture of the 4th rib at 90 mm and 212 mm from the sternocostal joint; Configuration 2: Segmental fracture of the 4th rib at 90 mm and 244 mm from the sternocostal joint; Configuration 3: Segmental fracture of the 4th and 5th rib at 90 mm and 112 mm, and 212 mm and 242 mm, respectively, from the sternocostal joint; Configuration 4: Segmental fracture of the 4th and 5th rib at 90 mm and 112 mm, and 244 mm and 283 mm, respectively, from the sternocostal joint; Configuration 5: Segmental fracture of the 3rd, 4th, and 5th rib at 64 mm, 90 mm and 112 mm, as well as 151 mm, 212 mm, and 242 mm, respectively, from the sternocostal joint; Configuration 6: Segmental fracture of the 3rd, 4th, and 5th rib at 64 mm, 90 mm and 112 mm, as well as 186 mm, 244 mm, and 283 mm, respectively, from the sternocostal joint

**Fig. 4:**
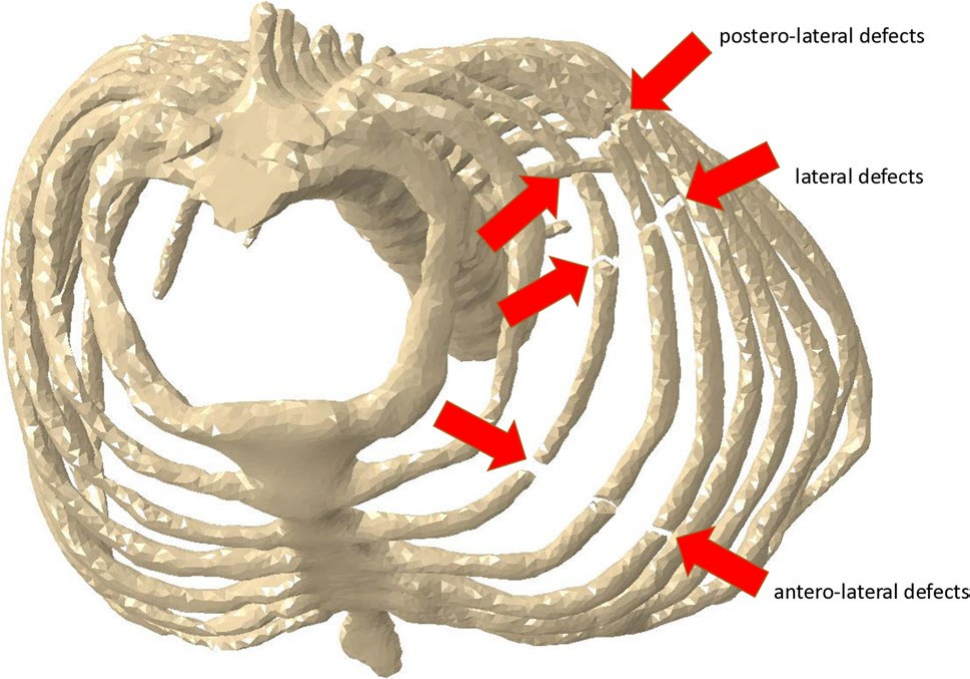
Visualization of the fracture localizations in top view

**Fig. 5:**
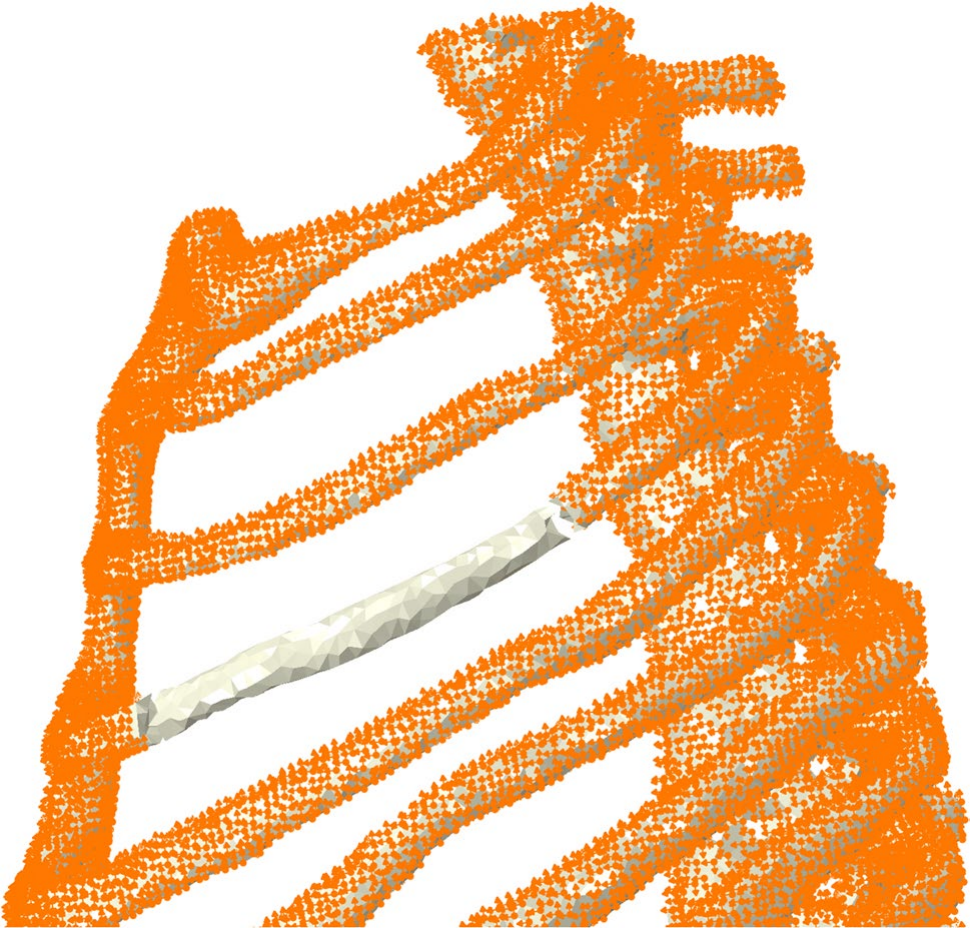
Schematic presentation of the predefined bony thoracic node displacement position indicated in orange for a short segmental fracture at the 4th left rib

1. We took the previous intact configuration to represent different fracture scenarios and removed elements from the bone thorax mesh according to predefined fracture model groups. These included segmental defects of different numbers of ribs and varying sizes of the segmental defects. This fracture model solely addressed Costae verae, and fractures affected the shaft segment according to the definition of the chest wall injury society [6]. In that model, short fracture segments affected the anterolateral and lateral subsegment, while long fracture segments affected the anterolateral and posterolateral subsegment of the corresponding ribs according to the definition of Ritchie et al. [24] (Fig. 3 and 4. We modified only Costae verae III to V. Fracture defects are modelled within the anterolateral shaft segments as well as the lateral shaft segments for the short defect and the posterolateral segments for the long defects. The total rib lengths were 263 mm, 326 mm, and 358 mm for rib III, IV, and V, respectively, with rib shaft lengths of 199 mm, 236 mm, and 246 mm for rib III, IV, and V, respectively. The defects were positioned along the ribs 64 mm, 90 mm, and 112 mm for rib III, IV, and V, respectively, from the sternum for the anterolateral defect; and 151 mm, 212 mm, and 242 mm for rib III, IV, and V, respectively, for the lateral defect (short defect) and 186 mm, 244 mm, and 283 mm for rib III, IV, and V, respectively, for the posterolateral defect (long defect). The short defect covered 33%, 37%, and 36% for rib III, IV, and V, respectively, of rib total length (ad sternum, including costal cartilage) or 44%, 52%, and 53% for rib III, IV, and V, respectively, of rib shaft length. The long defect covered 46%, 47%, and 48% for rib III, IV, and V, respectively, of rib total length (ad sternum, including costal cartilage) or 61%, 65%, and 70% for rib III, IV, and V, respectively, of rib shaft length.
2. In order to allow realistic segment movement, we removed the displacements on the bony segment nodes (Fig. 5). The segments can move freely but are still tied to the lung surface.
3. Changes in volume and pressure were measured during the entire simulation.

### Outcome measures of interest and statistical analysis

Based on the fracture configurations above, we measured the corresponding areas of the resulting segmental defects. We measured the sizes of each rib and the intercostal space between the defined fracture locations in coronal and sagittal reconstructions. Segmental fracture ares were calculated according to the geometrical form of a trapezoid.

The tidal volume was measured in the intact thorax model, and for each fracture configuration, the maximum volume change was between 0 kPa and − 0.3 kPa. The loss of tidal volume was defined as the difference in the maximum volume change of each fracture configuration compared to the intact model.

Further, the respiratory work was calculated for the intact model and each fracture configuration. The respiratory work is a frictionless, quasi-static force needed to realize a specific volume change at a certain pressure. The calculation is based on multiplying the negative pressure at the endpoint with the corresponding volume change. Consequently, it represents a physical reference value integral to the patient’s total muscle work. This physical value does not necessarily represent the patient’s respiratory work due to muscle contractions.

Statistical analysis was performed using GraphPad Prism (GraphPad Prism 10 for macOS, Version 10.2.3, GraphPad Holdings, LLC, San Diego, CA, USA). The impact of the size of the segmental defects on the tidal volume, loss of tidal volume, and respiratory work were assessed using correlation analyses. All p-values were two-tailed, and p-values < 0.05 were considered statistically significant.

## Results

**Fig. 6:**
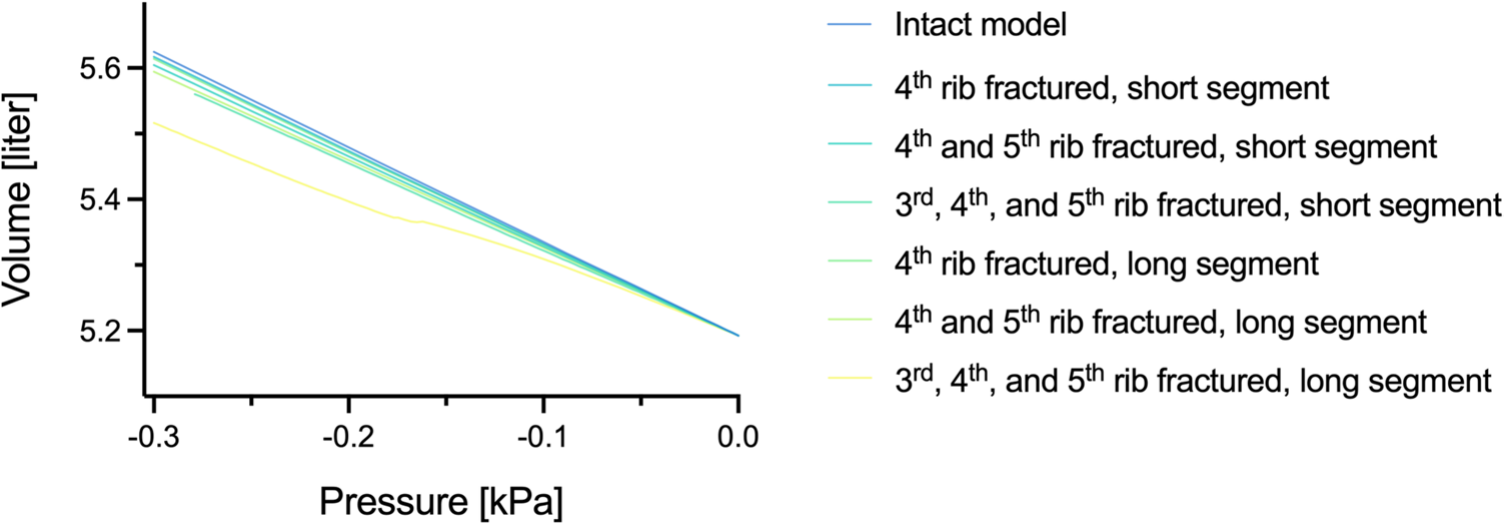
Pressure–volume diagram of the intact model and different fracture configurations

**Fig. 7:**
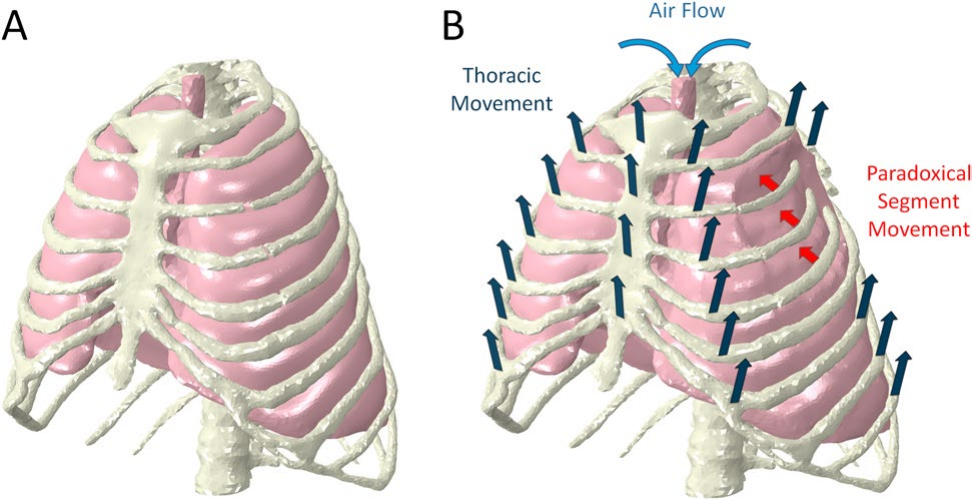
Paradoxical thoracic cage movement due to FC injury. **A** Expiration. **B** Inspiration. Please see the supplementary video link for the breathing cycle in motion: https://orthoload.com/biomechanics-of-the-unstable-thorax-respiratory-work-and-intrathoracic-volume-changes-in-segmental-rib-fractures/

The final model proved reliable physiological breathing patterns (Fig. 6) and showed clinically realistic changes in thoracic wall movement depending on the specific fracture pattern (Fig. 7).

The absolute values of the tidal volumes, absolute and relative loss of tidal volume, absolute respiratory work, and compensatory respiratory work needed to reach the tidal volume of an intact thorax are presented in Table 2.

**Table 2:** Absolute values of the outcome measures of interest

	Tidal volume [ml]	Absolute loss of tidal volume [ml]	Relative loss of tidal volume [%]	Respiratory work [J]	Compensatory respiratory work needed [%]
Intact model	432	n. a	n. a	0.1297	n. a
Configuration 1	424	7.7	1.78	0.1273	1.82
Configuration 2	421	11	2.54	0.1264	2.61
Configuration 3	412	20	4.65	0.1236	4.88
Configuration 4	402	30.4	7.03	0.1205	7.56
Configuration 5	367.7	64.4	14.91	0.1103	17.53
Configuration 6	324	108	25.06	0.0972	33.44

*n*. *a*. not applicable

**Fig. 8:**
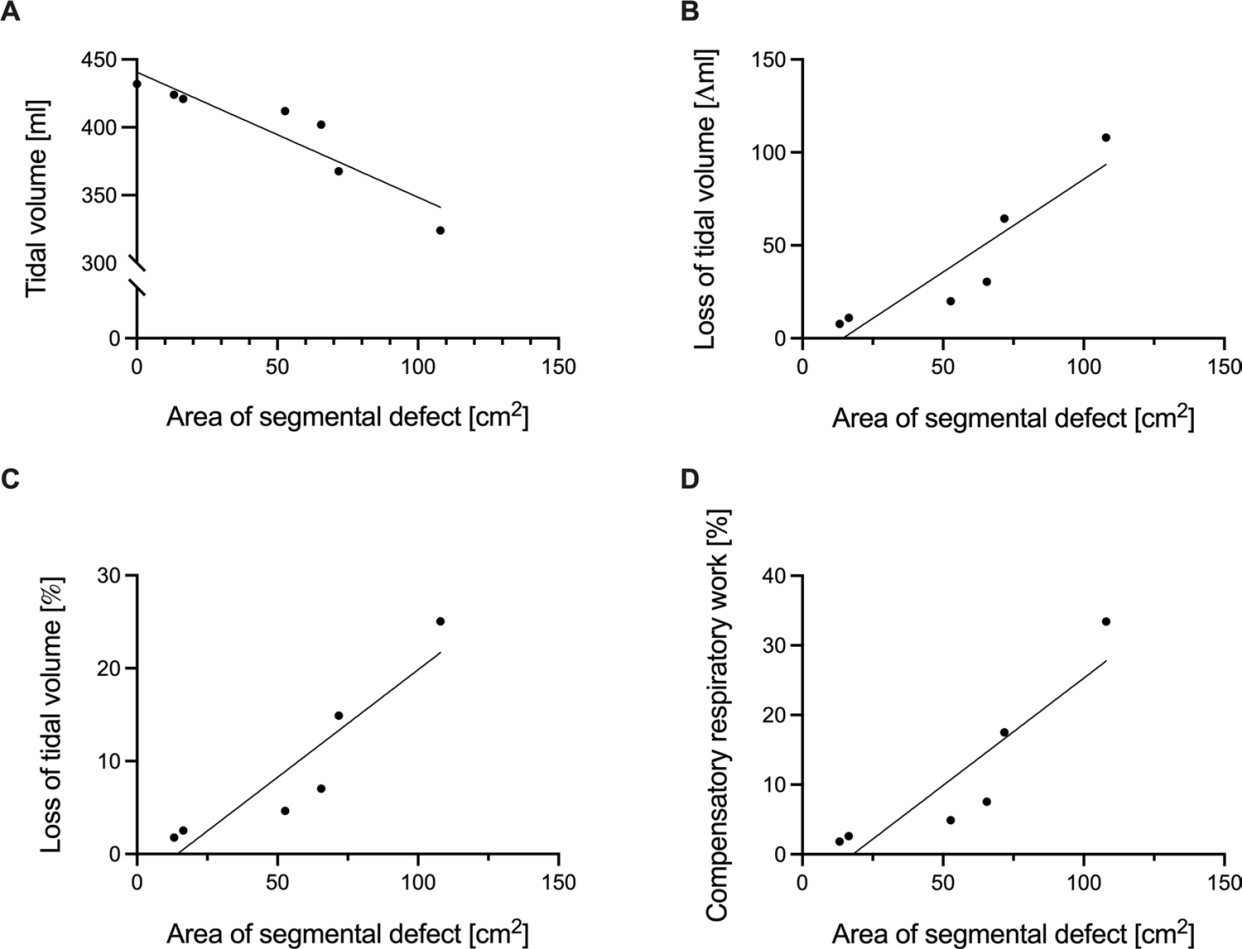
Impact of the segmental defect area on tidal volume and compensatory respiratory work required. The dependence of the absolute tidal volume on the segmental defect size is depicted in (**A**). Concerning the intact thorax model, (**B**) displays the absolute and (**C**) the relative loss of tidal volume loss in dependence on the segmental defect size. Last, (**D**) displays the calculated compensatory respiratory work needed depending on the segmental defect area

The correlation analysis showed a strong correlation between the size of the segmental defect and all outcome measures assessed. Larger segmental defects were significantly associated with lower tidal volume (R^2^ = 0.8523, *p* = 0.003; Fig. 8A), which corresponded to a higher absolute (R^2^ = 0.8445, *p* = 0.0096; Fig. 8B) and relative loss (R^2^ = 0.8444, *p* = 0.0096; Fig. 8C) of tidal volume compared to the intact thorax model. Last, larger segmental defects were associated with a higher need for compensatory respiratory work (R^2^ = 0.816, *p* = 0.0136; Fig. 8D).

## Discussion

In this study, we implemented a functional FE model of a thorax based on real-world injury data. With this model, we systematically analyzed fracture patterns of FC injuries. The corresponding results showed a strong negative correlation between the size of the flail segment and tidal volume. Further, the segment’s size correlated with the absolute and relative loss of tidal volume compared to the intact thorax, and compensatory respiratory work was needed.

Given that almost half of the falls from height and road traffic accidents are associated with severe blunt thoracic trauma [25, 26], FC injuries are regularly observed in MT patients [9]. Surgical treatment concepts are increasingly coming into focus due to the high morbidity and mortality of these injuries. Currently, 5.8% of all rib fractures [27, 28] and up to 16.7% of all FC injuries are surgically treated [28]. Studies observed that early surgical stabilization of FC injuries reduces LOS [14, 15, 29–32] and, during a stay on the ward, shortens the duration of invasive ventilation [33–36]. Further, surgical stabilization leads to fewer tracheotomies [29, 30, 37], a lower incidence of nosocomial pneumonia [33, 34], and ultimately, a lower mortality rate [30–32, 38, 39]. Recent analyses of the Traumaregister DGU underlined the superiority of a surgical stabilization of FC injuries compared to a conservative treatment [40–42]. However, some studies have reported contradictory observations regarding these outcomes [43–45].

The indications for surgery differed between previous study centers; these included: a) at least three fractured ribs with paradoxical movement [10], b) at least three consecutive ribs fractured in multiple places with a floating chest segment [11], or c) at least six fractured ribs leading to acute respiratory failure and need for mechanical ventilation [12]. The heterogeneity of indicating surgery results from a lack of evidence about fracture morphologies linked to paradoxical breathing patterns and the corresponding risk of respiratory insufficiency. Thus far, the definition of FC is solely based on the number of ribs involved in the flail segment, which does not consider the biomechanical characteristics of the fracture morphology.

The earliest approaches in designing an FE model of an anatomically correct human thorax date back to 1977 [46]. Since then, the fewest published models have focused on injured thoraces but on physiologic thorax movements during breathing [47]. Baumann et al. published an FE model based on standardized skeletal models [48]. Their data showed that the loss of chest wall stability (CWS) was highest for axial rotation and that the number of fractured ribs directly impacted the loss of CWS [48]. Further, they showed that CWS loss was 3% for a singular rib fracture and can be as high as 50% in complex fracture patterns. These observations match up with the data derived from our FE model. Overall, both models are consistent with observations from a cadaver study [49]. They connected three consecutive ribs with motion sensors, which were then osteotomized to induce an FC injury. They observed that FC injuries resulted in an 11.3-fold increase in sagittal plane motion compared to an intact thorax. This instability was reduced 2.1 times by stabilizing the FC segment via osteosynthesis. Marasco et al. [11] also assessed the impact of surgical stabilization using an FE model for posterior rib fractures. They observed that posteriorly located FC injuries tended to have a relevant posterior displacement due to the regular breathing motion. These fractures may be fixed using an intramedullary-placed bioresorbable screw.

Our model helps to understand the biomechanical impact of FC injuries. Based on real-world injury data, our model showed a correlation between the size of the flail segment and tidal volume and the need for compensatory respiratory work. As the size of the flail segment depended on the number of consecutively involved ribs and the length of the flail segment, this suggests a more complex definition of FC than currently used. Thus far, a prospective identification of patients at risk for paradoxical chest wall movements and respiratory failure is not feasible. However, this would be of utmost importance as the pulmonary derangements due to a respiratory insufficiency possibly resulting from the paradoxical chest wall movements play an essential role in the decision-making process when evaluating the indication for surgery [50]. Future FE models should implement different patients’ anatomy and simulate various FC configurations to prove our observations and gain further understanding. These observations must be linked to clinical data to depict a cut-off value of the flail area size and the risk for paradoxical breathing patterns. Additionally, studies may focus on the effects of fracture fixation using commonly used fixation systems on CWS and respiratory physiology. Thus far, no evidence-based recommendation indicates which ribs to fix in a flail segment to maximize stability while minimizing the procedure’s invasiveness.

Our study has a few limitations. First, based on CT data, this FE model is an idealized model of a previously injured thorax. During the modeling steps explained above, we had to set various assumptions and boundary conditions to achieve realistic breathing patterns and physiological respiratory mechanics. This was needed since the literature reports that material properties were highly heterogeneous. We aimed to reach a realistic tidal volume based on average spirometry data and the official recommendations for lung-protective ventilation but with minimal assumptions for diaphragmatic involvement. Second, the model is based on one CT data set. Accordingly, it represents a specifically selected but anatomically correct and typical thorax of one specific individual of a larger cohort of MT patients, which may not allow us to draw a universally valid conclusion. Therefore, we recommend that future authors segment various anatomic configurations before implementing the fractures to exclude the impact of patients’ anatomy on the results of the FE model. Last, we limited the analyzed fracture configurations to six representative scenarios on one specific side. Although our data suggest a correlative impact of the size of the flail segment on the assessed outcome measure, future studies should assess a wider variety of fracture configurations. Of interest would be segmental injuries that do not fulfill the FC definition (e.g., two ribs with a very long fracture segment and FC injuries with a concise fracture segment). These configurations at the edge of the current FC definition are of utmost interest to improve the definition and gain further understanding of FC mechanics to prospectively identify patients at risk that benefit from early surgical stabilization. However, this is the first study to assess various standardized fracture configurations on respiration with active breathing in a simulation based on CT data from an FC patient. Our data shed light on the persisting lack of evidence regarding the indication for FC stabilization based on the definition of this injury.

## Conclusion

This study presents an FE analysis of the thorax of a patient who presented to our clinic as an MT patient with an FC injury. The model fulfills physiologic breathing patterns in a simulation of an actively breathing patient and realistically displays the paradoxical movement of an FC injury. Our simulation of different fracture configurations showed that the number of consecutive ribs involved in the flail segment and the length of the flail segment significantly impacted active breathing. This suggests that the indication for FC stabilization should not be solely based on the current definition of FC, as consecutively needed compensatory breathing work resulting from the loss of tidal volume correlates with the size of the flail segment. From a clinical perspective, we need to consider the impact of the flail segment size on the risk of paradoxical breathing. Considering this, we strongly recommend implementing these findings into the definition of FC to indicate surgical stabilization based on biomechanical observations more accurately. Still, our findings must be corroborated with future studies and correlated with clinical observations.

## Supplementary Information

Below is the link to the electronic supplementary material.

Supplementary file1 (DOCX 12 KB)

## Data Availability

Please see the supplementary video link for the respiratory cycle in motion: https://orthoload.com/biomechanics-of-the-unstable-thorax-respiratory-work-and-intrathoracic-volume-changes-in-segmental-rib-fractures/ All data related to the simulation model can be obtained upon justified request via https://orthoload.com/contact/ or directly from the authors.
