# Supplementary material for: Biomechanics of flail chest injuries: tidal volume and respiratory work changes in multiple segmental rib fractures
Source: Eur J Trauma Emerg Surg. 2025 Jan 17;51(1):25. doi: 10.1007/s00068-024-02754-x (PMC11742335; doi:10.1007/s00068-024-02754-x)
Supplement: Supplementary file 1 — Supplementary file1 (DOCX 12 KB) [file 68_2024_2754_MOESM1_ESM.docx]

Please see the [supplementary video link for the breathing cycle in motion](https://orthoload.com/biomechanics-of-the-unstable-thorax-respiratory-work-and-intrathoracic-volume-changes-in-segmental-rib-fractures/): https://orthoload.com/biomechanics-of-the-unstable-thorax-respiratory-work-and-intrathoracic-volume-changes-in-segmental-rib-fractures/
